# Using breeding and quantitative genetics to understand the C_4_ pathway

**DOI:** 10.1093/jxb/erab486

**Published:** 2021-11-08

**Authors:** Conor J C Simpson, Gregory Reeves, Anoop Tripathi, Pallavi Singh, Julian M Hibberd

**Affiliations:** 1 Department of Plant Sciences, University of Cambridge, Cambridge, UK; 2 University of Edinburgh, UK

**Keywords:** C_4_ photosynthesis, natural variation, hybridization, mapping population designs

## Abstract

Reducing photorespiration in C_3_ crops could significantly increase rates of photosynthesis and yield. One method to achieve this would be to integrate C_4_ photosynthesis into C_3_ species. This objective is challenging as it involves engineering incompletely understood traits into C_3_ leaves, including complex changes to their biochemistry, cell biology, and anatomy. Quantitative genetics and selective breeding offer underexplored routes to identify regulators of these processes. We first review examples of natural intraspecific variation in C_4_ photosynthesis as well as the potential for hybridization between C_3_ and C_4_ species. We then discuss how quantitative genetic approaches including artificial selection and genome-wide association could be used to better understand the C_4_ syndrome and in so doing guide the engineering of the C_4_ pathway into C_3_ crops.

## Teaser

We review literature relevant to forward genetics and consider when and how it could be applied to better understand C_4_photosynthesis.

## Introduction

Photosynthetic plants provide humanity’s food, many textiles, and building materials, and represent the source of numerous medicines and fuels. Understanding how improvements in photosynthesis could be achieved therefore has the potential to impact many aspects of human life. Photosynthesis requires the enzyme Rubisco to fix atmospheric carbon dioxide (CO_2_) into 3-phosphoglycerate (Calvin and Benson, 1948). Species that only use Rubisco for carbon fixation are known as ‘C_3_’ plants, as 3-phosphoglycerate contains three carbon atoms. Rubisco, however, is also able to react with oxygen in addition to CO_2_. This oxygenation reaction produces the toxic molecule 2-phosphoglycolate, which must be metabolized and recycled via the photorespiratory cycle. Photorespiration leads to loss of carbon fixed by Rubisco and release of ammonia from amino acids at the expense of both ATP and reducing power (Bowes *et al.*, 1971). Rates of photorespiration typically increase at higher temperatures because, under these conditions, the oxygenation reaction of Rubisco is favoured (Portis and Parry, 2007), but photorespiration can also increase during periods of drought when stomatal closure limits CO_2_ supply to the Rubisco active site. In extreme conditions, photorespiratory rates can use ~25% of photosynthetic outputs (Sharkey, 1988).

**Fig. 1.**
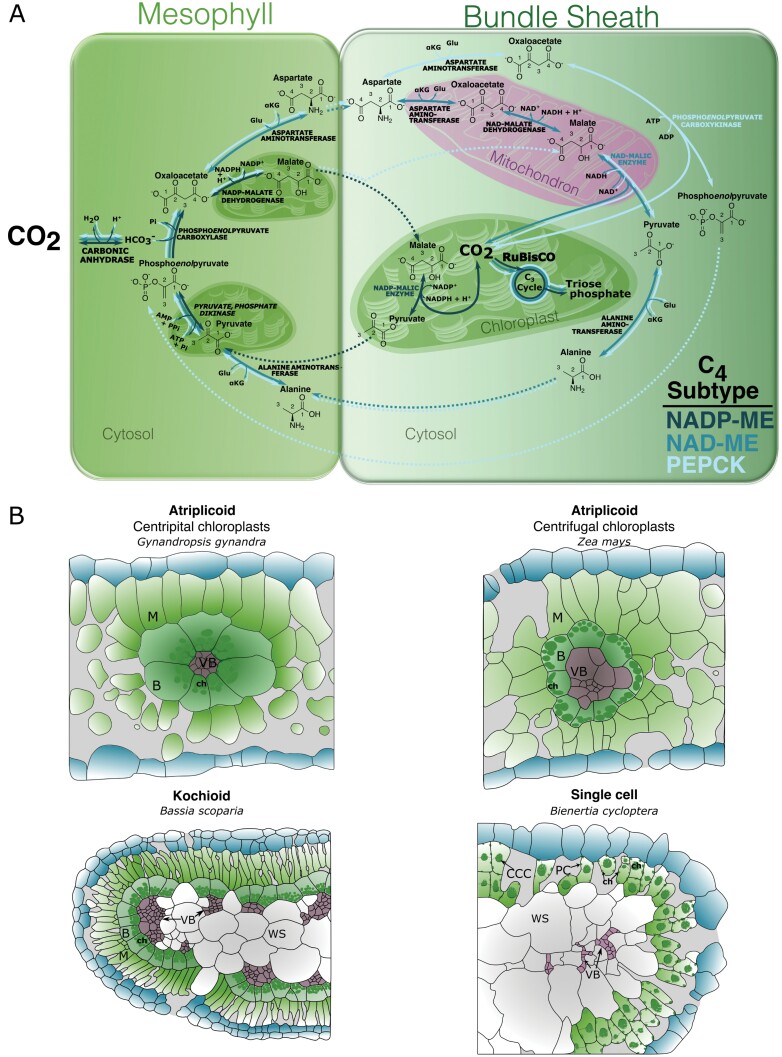
Natural variation in C_4_ biochemistry and anatomy. (A) An overview of C_4_ biochemical subtypes. Although all forms of two-celled C_4_ photosynthesis involve initial CO_2_ fixation to generate four-carbon intermediates in mesophyll cells and diffusion to bundle sheath cells, the method of decarboxylation to create a high-CO_2_ environment around Rubisco varies between C_4_ species. Solid and dashed lines show enzymatic and diffusion steps of the C_4_ pathway, respectively. (B) Examples of leaf anatomies seen in C_4_ species. Exemplar species that use each anatomical variant are shown below each type. Many more anatomical types have been described, which suggests that multiple leaf morphologies can facilitate the C_4_ pathway. Abbreviations: M, mesophyll; B, bundle sheath; VB, vascular bundle; CCC, central cytoplasmic compartment; PC, peripheral chloroplast; WS, water storage cell; ch, chloroplast.

Land plants have evolved two carbon-concentrating mechanisms to reduce photorespiration. These are termed Crassulacean acid metabolism (CAM) and C_4_ photosynthesis. Whilst in both cases rates of photorespiration are reduced because compared with the C_3_ state, ~10-fold higher concentrations of CO_2_ are supplied to Rubisco, CAM and C_4_ species use temporal and spatial systems, respectively. It is estimated that the C_4_ pathway has evolved independently from C_3_ ancestors at least 60 times to yield numerous phenotypes that concentrate CO_2_ around Rubisco (Sage *et al.*, 2011). In all cases, in the C_4_ leaf Rubisco-dependent fixation of CO_2_ takes place in a specific compartment supplied with high concentrations of CO_2_ such that the oxygenase activity of Rubisco is almost completely abolished (Fig. 1A). In most C_4_ species, photosynthesis is compartmented between two cell types so that they are unified by a general pathway in which CO_2_ is converted to bicarbonate (HCO_3_^–^) by carbonic anhydrase (CA) in mesophyll cells, and then combined with the 3-carbon molecule phospho*enol*pyruvate (PEP) by the enzyme phospho*enol*pyruvate carboxylase (PEPC) into the 4-carbon molecule oxaloacetate (Fig. 1A). Oxaloacetate is then either reduced to malate or transaminated to aspartate. After diffusing to an adjacent cell layer such as the bundle or mestome sheath, malate or aspartate are decarboxylated such that high concentrations of CO_2_ accumulate around Rubisco and so allow high rates of carboxylation (Fig. 1A). Finally, in species that use NAD-dependent malic enzyme (NAD-ME) or NADP-ME to release CO_2_ around Rubisco, the 3-carbon molecule produced from decarboxylation is regenerated to PEP in mesophyll cells by pyruvate orthophosphate dikinase (PPDK) to continue the cycle (Fig. 1A).

Traits underpinning C_4_ photosynthesis vary widely between species (Edwards and Voznesenskaya, 2011; Furbank, 2011; Sage and Stata, 2015; Sedelnikova *et al.*, 2018). This interspecific variation in C_4_ traits includes differences in leaf anatomy, cell biology, and biochemistry, as well as the patterns of gene expression that determine these characteristics. For example, the cell types and arrangement of veins used by C_4_ species vary between lineages that have independently evolved the pathway (Fig. 1B). At least nine anatomical types have been described in the grasses (Poaceae) (Edwards and Voznesenskaya, 2011). Examples of this variation include in the number of layers of mestome and/or bundle sheath cells, and whether Rubisco is compartmented into the bundle or the mestome sheath. Although much of this variation associated with C_4_ photosynthesis is found in lineages that are separated by deep evolutionary time, Kranz anatomy also differs in species within families including the Amaranthaceae (Kadereit *et al.*, 2003; Muhaidat *et al.*, 2007; Sage, 2016), Asteraceae (Peter and Katinas, 2003), Cleomaceae (Koteyeva *et al.*, 2011), Portulaceae (Voznesenskaya *et al.*, 2017), and Poaceae (Ohsugi and Murata, 1985; Edwards and Voznesenskaya, 2011). Of the ~8100 C_4_ species defined to date, six operate the C_4_ pathway in a single cell (Fig. 1B). In these single-celled C_4_ species, the pathway is distributed between separate populations of chloroplasts such that the cell biology of these species has been modified compared with the C_3_ state. However, modifications to the cell biology of C_4_ leaves is not restricted to these single-cell species. In C_4_ species that separate photosynthesis between two cell types, plasmodesmatal frequency is increased compared with the C_3_ state (Botha, 1992; Danila *et al.*, 2016). Some lineages contain suberin in the bundle sheath cell wall whilst others do not (Mertz and Brutnell, 2014), and whilst some C_4_ lineages arrange chloroplasts in bundle sheath cells centripetally, others do this centrifugally with respect to the veins (Edwards and Voznesenskaya, 2011).

Lastly, soon after the discovery of C_4_ photosynthesis, differences in the biochemistry of the pathway were discovered among C_4_ species (Hatch *et al.* 1975). These different pathways were termed C_4_ ‘subtypes’ due to the fact that decarboxylation is associated with three separate C_4_ acid decarboxylases, NADP-ME, NAD-ME, and phospho*enol*pyruvate carboxykinase (PEPCK). Although there is growing support for the notion that species can modify the extent to which each C_4_ acid decarboxylases is engaged (Omoto *et al.*, 2012; Sharwood *et al.*, 2014; Sales *et al.*, 2018), the differences in biochemistry associated with the subtypes exemplify the fact that the C_4_ pathway is a convergent phenomenon, and that its operation varies between species.

The differences in leaf anatomy, cell biology, and biochemistry between independent C_4_ lineages have frequently been summarized (Edwards and Voznesenskaya, 2011; Sage, 2016). In contrast, there have been fewer recent attempts to synthesize the literature relating to forced hybridizations between C_3_ and C_4_ species. Studies have included somatic hybridizations of phylogenetically distant C_3_ and C_4_ plants, as well as sexual hybridizations of congeneric species. Whilst these wide hybridizations have provided insight into the extent to which C_4_ traits can be maintained and inherited in C_3_ species, a growing body of evidence documents variation in C_4_ traits within a species. We summarize examples of this work and suggest that there are opportunities to use quantitative trait mapping to better understand the C_4_ pathway. Not only could these classical approaches provide insight into the evolution and genetic basis of C_4_ photosynthesis, they may also inform efforts to engineer more efficient C_3_ crops.

## Somatic hybridization of C_3_ and C_4_ species

Approaches such as protoplast fusion allow somatic or asexual hybridization. Protoplasts from somatic cells from separate species are fused and regenerated into hybrid plants (Carlson *et al.*, 1972; Evans, 1983). In many cases, asexual hybridization can lead to fertile hybrids between species that are considered sexually incompatible. Attempts to form hybrids via somatic hybridization of C_3_ rice (*Oryza sativa*) and other C_4_ grasses have been moderately successful. Terada *et al.* (1987) produced somatic hybrids between rice and C_4_*Echinochloa oryzicola* that were morphologically different from either parent. Some contained 60 chromosomes which corresponded to the full hybrid complement, but plants developed necrosis and died before forming roots. Moreover, rice and C_4_*Panicum maximum* (now *Megathyrsus maximus*) were successfully fused to form hybrids with abnormal floral structures with lowered fertility (Xin *et al.*, 1997). In all, 28 hybrids flowered but only five set fertile seed. To our knowledge, this work has never been repeated.

There have also been attempts to form hybrids between wheat and C_4_ grasses. A cell suspension of Trititrigia (a perennial hybrid of *Triticum durum* and *Thinopyrum intermedium*) was hybridized with maize (Wang *et al.*, 1993; Wang and Niizeki, 1994). Plants that regenerated were aneuploids carrying incomplete sets of chromosomes from both species. Although the progeny were not full hybrids, this study demonstrated that after asexual hybridization, maize and *Triticum* chromosomes were not eliminated during successive cell divisions despite the uniparental genome elimination that occurs when both species are hybridized sexually (Laurie and Bennett, 1986, 1989; Laurie *et al.*, 1990). Szarka *et al.* (2002) fused a cell suspension of an albino maize mutant with wheat protoplasts. Plants that regenerated resembled maize but were green, indicating that photosynthesis from wheat rescued the albino phenotype in maize. Cytological observations showed the plants had all parental chromosomes, but no morphological traits associated with C_4_ photosynthesis were detected and, although the plants produced male and female flowers, all were sterile (Szarka *et al.* 2002). Independently, Xu *et al.* (2003) reported wheat–maize hybrids that contained nuclear and mitochondrial genomes of both species but plastid DNA only from wheat. These somatic hybrids resembled wheat and, although many flowered, they were all sterile. This may have been due, at least in part, to the fact that the wheat and maize cell suspension cultures had chromosomal aberrations prior to fusion. Thus, taken as a whole, work on asexual hybridization of C_3_ and C_4_ cereals indicates that chromosomes of both photosynthetic types are stable in fused cells. However, in reports such as those from Xu *et al.* (2003) and Szarka *et al.* (2002), plants were not viable after transfer from tissue culture. In contrast, sexual hybridization of closely related C_3_ and C_4_ species has in some cases allowed production of fertile plants and their progeny assessed over multiple generations. We address this next.

## Sexual hybridization of C_3_ and C_4_ species

**Fig. 2.**
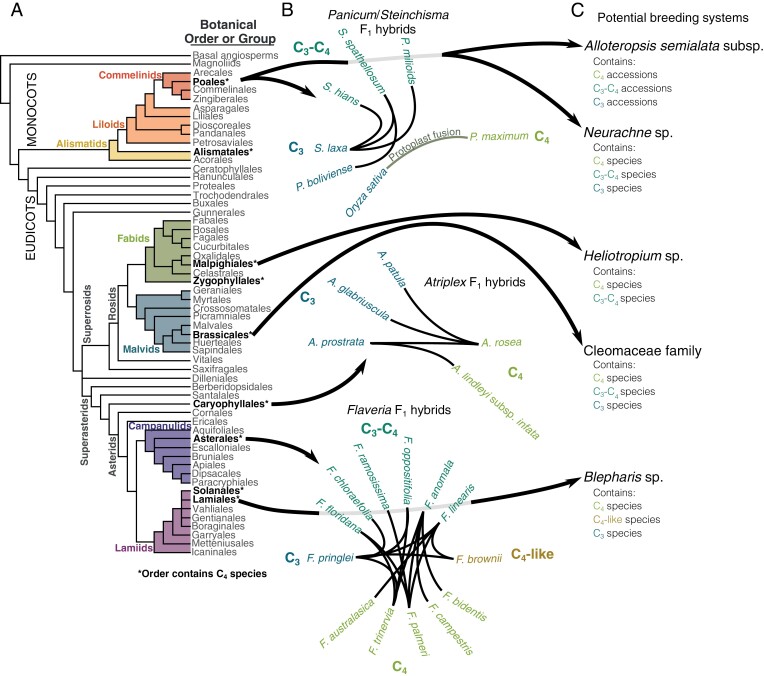
Examples of successful as well as potential hybridizations between C_3_ and C_4_ species. (A) Phylogenetic reconstruction of the orders constituting flowering plants according to The Angiosperm Phylogeny Group (2016). Orders containing C_4_ lineages are shown in bold. (B) Exemplar hybridization webs that have resulted in successful F_1_ hybrids between C_3_, C_4_, and C_3_–C_4_ intermediate photosynthetic types. (C) Taxa that contain closely related C_3_, C_4_, or C_3_–C_4_ intermediate species or accessions for which hybridization has not been reported, but may be possible. These groups are potential systems where C_4_ genes could be mapped. Arrows from the phylogenetic tree indicate from which order the plant species originate (B, C).

A number of taxa containing either congeneric C_3_ and C_4_ species or C_3_, C_3_–C_4_ intermediates, and C_4_ species have been successfully hybridized (Fig. 2A, B). Although the outcome of these analyses varied, whilst wholesale transfer of C_4_ traits have not been reported in some instances, specific traits were introgressed into a C_3_ background. For example, crosses between C_4_*Atriplex rosea* and C_3_*Atriplex prostrata* (formerly *A. patula* ssp. *hastata* and *A. triangularis*, respectively), C_3_*A. rosea* and C_3_*A. glabriuscula* have been made (Björkman *et al.*, 1969; Nobs *et al.*, 1970). Populations derived from such crosses were progressed and C_4_-like characteristics assessed (Björkmann *et al.*, 1971). Among 200 F_3_ individuals screened for the CO_2_ compensation point, 178 individuals showed values similar to the C_3_ parent, 19 showed intermediate phenotypes, and three were similar to the C_4_ parent (Björkman *et al.*, 1969). Thus, in a small number of individuals, it appears that crossing was able to integrate loci associated with the compensation point. When F_1_ derived from a C_4_*A. rosea*×C_3_*A. patula* hybridization were backcrossed to C_4_*A. rosea*, these BC_1_ offspring segregated for either C_4_ or C_3_ photosynthesis, with only two individuals showing C_4_ photosynthesis (Rikiishi *et al.*, 1988), suggesting dominance towards a C_3_ state in this hybrid combination. In these reports above, no F_1_ individual, nor any within segregating F_2_ and F_3_ populations, showed a full transfer of C_4_ photosynthesis. More recently, F_2_ individuals derived from a resynthesized C_4_*A. rosea*×C_3_*A. prostrata* cross showed large variation in leaf anatomy and nearly intermediate CO_2_ compensation points, but individuals in the F_3_ generation seemed to revert to C_3_-like values (Oakley *et al.*, 2014). Hybrids have also been made between C_3_ and C_4_-like species of *Flaveria* (Apel *et al.*, 1988; Cameron *et al.*, 1989) and C_3_–C_4_ intermediate and C_4_*Flaveria* species (Brown et al., 1986, 1992). Significant F_1_ sterility was encountered (Brown and Bouton, 1993) but F_2_ were obtained and, although they possessed continuous variation with regard to C_4_ leaf anatomy and carbon isotope discrimination characteristics, it was skewed away from the mid-parental mean towards a C_3_ or C_3_–C_4_ phenotype. This would indicate dominance deviation towards a C_3_ phenotype despite the presence of genes that allow C_4_ photosynthesis. In F_1_ hybrids derived from a C_3_×C_4_-like *Flaveria* cross, enzyme activities of PEPC, PPDK, and NADP-ME were skewed towards those associated with C_3_ photosynthesis, but C_4_-like activities were reported for NADP-malate dehydrogenase (Holaday *et al.*, 1988), indicating that incomplete dominance for certain genes may exist while others show dominant activity patterns. In summary, although many C_3_×C_4_ hybrids in the dicotyledons showed reduced fertility and limited penetrance of C_4_ traits, these studies also indicate that aspects of C_4_ photosynthesis are heritable in a C_3_ background. As many other closely related C_3_ and C_4_ species exist (Fig. 2C), it is possible that additional stable hybrids could be generated that exhibit increased genomic stability and/or better trait segregation between the C_3_, C_3_–C_4_, and C_4_ types. Hybrids between different C_4_ decarboxylation subtypes may also be possible. Closely related species such as *Blepharis cilaris* and *Blepharis attenuata* that use NAD-ME and NADP-ME, respectively, have been described (Akhani *et al.*, 2008). To our knowledge, whilst no hybrids have been reported in *Blepharis*, natural hybrids between *Cynodon dactylon* (NAD-ME) and *Chloris* sp. (PEPCK) display intermediate activities of NAD-ME and PEPCK (Prendergast, 1987).

C_3_–C_4_ hybrids have been generated in the grasses by two broad approaches. First, as with dicotyledons, congeners using either C_3_ or C_3_–C_4_ photosynthesis have been crossed. Second, much wider crosses of distantly related species have been performed. Examples of crosses within a genus include C_3_ and C_3_–C_4_ intermediate *Steinchisma* (formally *Panicum*) species from the Poaceae (Bouton *et al.*, 1986; Brown *et al.*, 1986; Sternberg *et al.*, 1986). F_2_ and F_5_ individuals derived from hybridization of *Steinchisma milioides* (C_3_–C_4_) and *Steinchisma laxum* (C_3_), or *S. spathellosum* (C_3_–C_4_) and *S. boliviense* (C_3_) exhibited intermediate leaf morphologies, CO_2_ compensation points, and δ^13^C values. Also within the Poaceae, C_3_ and C_4_ accessions of *Alloteropsis semialata* have been hybridized, producing plants with intermediate anatomical traits as well as C_4_ gene expression (Bianconi *et al.*, 2021, Preprint). Thus, in these hybridizations, some traits important for C_4_ photosynthesis could be introduced into an otherwise C_3_ leaf. A variety of attempts at wide hybridization have also been reported. For example, although maize pollen germinates and fertilizes the ovule of wheat to form zygotes containing a full haploid set of each parental genome (Laurie and Bennett, 1986), these hybrids were unstable and after three rounds of mitotic cell divisions during embryogenesis all maize chromosomes were lost (Laurie and Bennett, 1986, 1989). In contrast, after hybridization of oat and pearl millet (*Pennisetum glaucum*) (Gernand *et al.*, 2005; Ishii *et al.*, 2010), some oat embryos contained all pearl millet chromosomes, and embryo rescue allowed hybrids possessing the haploid genomes of both species to be obtained (Ishii *et al.*, 2013). It appears that the pearl millet chromosomes had incorporated centromeric oat histones (Ishii *et al.*, 2015), but these haploid oat–millet F_1_ hybrids developed necrosis and died. This may have been caused by incompatibility between the species or non-ideal tissue culture conditions. Crosses between wheat and grain pearl millet (*Pennisetum americanum*) or oat and maize both allowed individual chromosomes from one species to be incorporated into the other. In the case of wheat and grain pearl millet from 958 hybridizations, one wheat plant carrying an additional pearl millet chromosome was identified (Ahmad and Comeau, 1990). Although this chromosome was maintained until flowering, it was not detected in the next generation. Thus, wheat–pearl millet hybrids may be more stable than wheat–maize hybrids, but problems maintaining chromosomes from both parents still appear to exist. Unlike wheat–maize hybrids, maize chromosomes have successfully been integrated into oat. This allowed the synthesis of so-called oat–maize chromosome addition lines that stably inherit single chromosome pairs from maize (Kynast *et al*., 2001, 2004). As with the pearl millet–oat crosses (Ishii *et al.*, 2015), stability of the oat–maize addition lines appears to be mediated by incorporation of centromeric oat histones into the maize chromosomes such that proper chromosomal segregation can take place during mitosis (Jin *et al.*, 2004; Wang *et al.*, 2014). In some maize–oat lines, C_4_ characteristics such as abundant transcripts of *PEPC* or C_4_-like bundle sheath cell size and vein spacing were detected (Tolley *et al.*, 2012).

In summary, the findings based on wide hybridization of maize and oat indicate that breeding offers a possible route to incorporate some C_4_ traits into C_3_ crops without prior knowledge of the underlying genetics. Although additional parental combinations may exist that allow greater trait stability in progeny, this approach has not yet allowed loci controlling C_4_ traits to be identified. In contrast, quantitative variation in C_4_ characteristics within a C_4_ species would allow trait mapping, and there is increasing evidence that this could be informative.

## Intraspecific variation in C_4_ photosynthesis

As PEPC discriminates less than Rubisco against the ^13^C isotope, a stronger C_4_ cycle leads to lower incorporation of ^13^C into tissue and so less negative δ^13^C values (Leary, 1988). Intraspecific variation in δ^13^C has been reported in maize and *Gynandropsis gynandra* (Voznesenskaya *et al.*, 2007; Kolbe and Cousins, 2018; Kolbe *et al.*, 2018; Reeves *et al.*, 2018; Twohey *et al.*, 2019). To our knowledge, the extent to which this variation in C_4_ efficiency is caused by differences in Kranz anatomy, cell biology, or C_4_ biochemistry has not been determined but, as summarized next, variation in some of these traits within a species has been reported. This includes variation in vein density in maize (Yabiku and Ueno, 2017; Kolbe and Cousins, 2018) as well as bundle sheath cell size in *Alloteropsis semialata* (Lundgren *et al.*, 2016) and *G. gynandra* (Reeves *et al.*, 2018). Thus, natural variation in Kranz anatomy is found within species of C_4_ monocotyledons and dicotyledons. Statistical modelling suggests that evolution of enlarged bundle sheath cells and vein density were among the first changes to occur during the transition from C_3_ to C_4_ photosynthesis (Williams *et al.*, 2013), and phylogenetic reconstructions reveal that these changes probably happened in response to reduced water availability (Edwards and Smith, 2010). As bundle sheath cell size and vein density were found to be correlated with water use efficiency in maize (Yabiku and Ueno, 2017) and *G. gynandra* (Reeves *et al.*, 2018), it is possible that analysis of C_4_ accessions adapted to different water availabilities will allow additional examples of intraspecific variation in Kranz anatomy to be identified.

While bundle sheath cells are always greener in C_4_ compared with C_3_ species, the proportion of leaf tissue allocated to bundle sheath cells compared with the mesophyll cells can be caused by either increased bundle sheath cell size or vein density (Sedelnikova *et al.*, 2018). Interestingly, within *G. gynandra*, these characteristics co-vary and correlate negatively with one another (Reeves *et al.*, 2018). In addition to variation in Kranz anatomy in a species, there is also evidence that the cell biology of C_4_ leaves can differ. For example, some accessions of *Panicum coloratum* possess a suberized bundle sheath whilst others do not (Ohsugi and Murata, 1985). There is also variation in chloroplast organization, with some accessions arranging chloroplasts centrifugally and others centripetally compared with veins (Ohsugi and Murata, 1985). Interestingly, *Cynodon dactylon*, an NAD-ME subtype with centripetal chloroplasts and a suberized bundle sheath, hybridizes naturally with *Chloris* that uses PEPCK as the primary C_4_ acid decarboxylase, has centrifugally arranged chloroplasts, and no suberization of the bundle sheath (Prendergast, 1987). F_1_s demonstrated intermediacy for these traits (Prendergast, 1987). Thus, these species offer an interesting system to study regulators of bundle sheath cell biology.

To our knowledge, there are no clear examples of quantitative variation in the extent to which accessions of an individual C_4_ species use the various C_4_ acid decarboxylases. However, there are two reasons to consider this likely. First, in 26 founder lines of a maize multiparent population, variation in the activities of C_4_ enzymes has been reported (McMullen *et al.*, 2009; Kolbe *et al.*, 2018). As the founders show differences in enzyme activity, it is likely that lines of the mapping population possess similar variation. Accessions of *A. semialata* (Dunning *et al.*, 2017) and *G. gynandra* (Reeves *et al.*, 2018) demonstrate differences in transcript abundance and so it appears likely that these species will also demonstrate variation in activity of C_4_ acid decarboxylases. Second, the extent to which the different C_4_ acid decarboxylases are engaged can vary with the environment. For example, in *G. gynandra* and maize, increased abundance of transcripts encoding C_4_ enzymes did not correlate with photosynthetic efficiency (Kolbe and Cousins, 2018; Reeves *et al.*, 2018) but in *G. gynandra* they were associated with increased water use efficiency. Additionally, the PEPCK subtype is considered more efficient under lower levels of light since it theoretically requires fewer quanta of light per CO_2_ molecule fixed (Furbank, 2011; Yin and Struik, 2020). Consistent with this, sugarcane (*Saccharum offiniarum*) and maize which predominantly use NADP-ME showed lower and higher activities of NADP-ME and PEPCK, respectively, after either shade or salt stress (Omoto *et al.*, 2012; Sharwood *et al.*, 2014; Sales *et al.*, 2018). Increased CO_2_ leakage from bundle sheath cells has also been reported, and it has been proposed that this is caused by increased use of cytosolic PEPCK compared with the chloroplastic NADP-ME (Sales *et al.*, 2018). If populations of these species have become reproductively isolated in habitats with distinct light supplies, differences in subtype preference may have evolved. Thus, C_4_ traits ranging from discrimination against δ^13^C, C_4_ leaf anatomy, bundle sheath cell biology, and C_4_ transcript abundance have been documented within a species. In each case, breeding and quantitative genetics offer an opportunity to identify loci controlling these traits. Within this context, we next assess opportunities associated with quantitative genetics to better understand C_4_ photosynthesis.

## Quantitative genetics and C_4_ photosynthesis

Quantitative genetics allow traits exhibiting continuous variation to be linked to genomic regions termed quantitative trait loci (QTL). Advances in high-throughput phenotyping relevant to photosynthetic performance (reviewed by Choudhury *et al.*, 2019; van Bezouw *et al.*, 2019) mean that quantitative genetics now offers a path to dissect the genetics underlying photosynthesis.

**Fig. 3.**
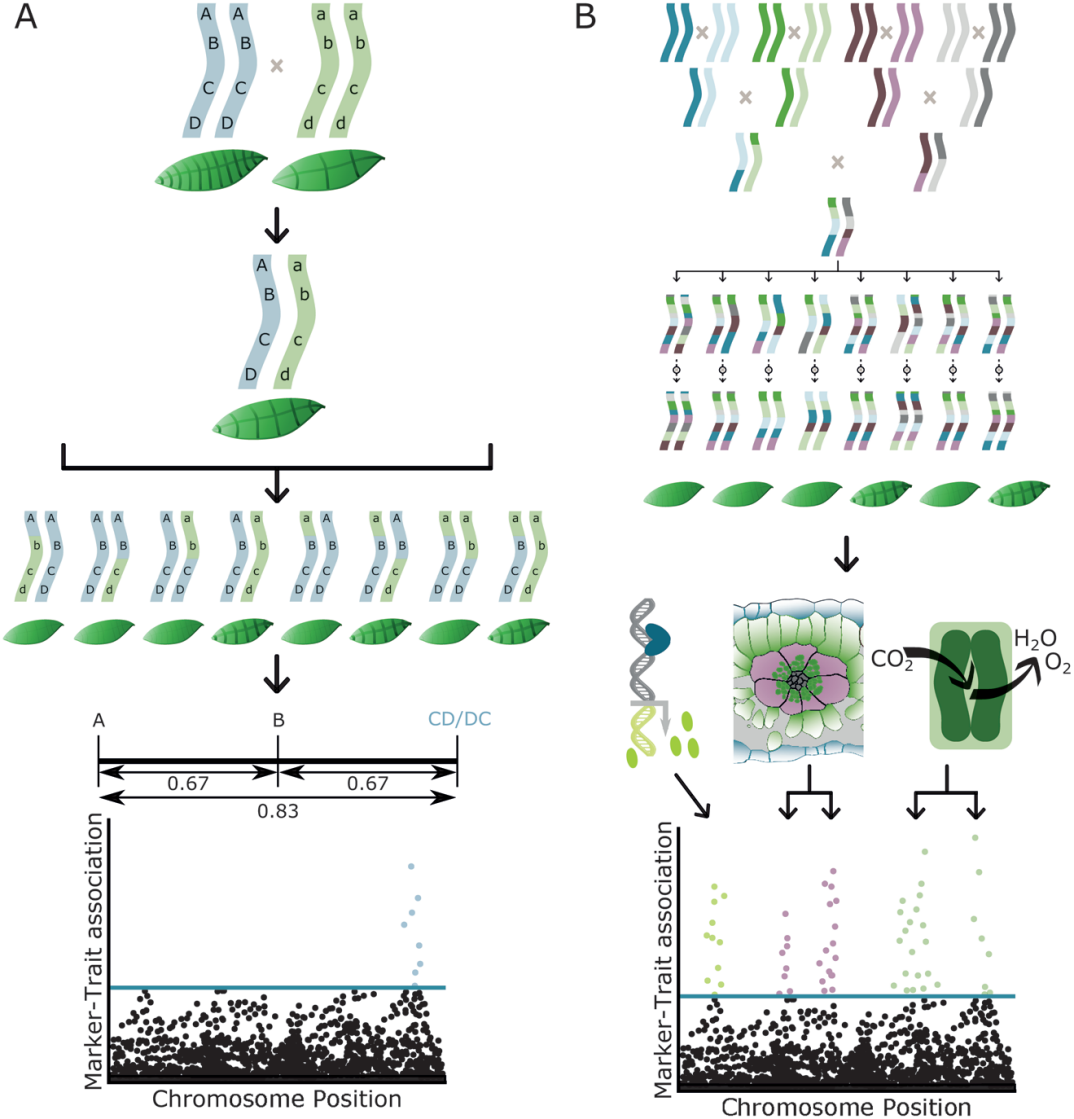
Quantitative genetics in the context of C_4_ photosynthesis. (A) A schematic for QTL mapping of leaf anatomical traits. Two homozygous parents, genotyped for four markers, A, B, C, and D, and differing in vein density are hybridized and advanced to form a bi-parental population that can be used to identify QTL associated with vein density (here located near markers C and D). Numbers show recombination fractions, which are used to position the QTL relative to flanking markers. (B) Population structure of a MAGIC pedigree followed by four generations of intercrossing and self-fertilization. Progeny contain more genetic variation than that derived from a bi-parental design. Hypothetical plot showing how QTL associated with individually mapped C_4_ phenotypes such as gene expression, bundle sheath cell size, or gas exchange parameters (e.g. stomatal conductance, CO_2_ assimilation, etc,) can be mapped with one population.

Traditional QTL mapping requires a linkage map (or genetic map) to order loci. Using a population derived from two parents that differ in a trait of interest, associations between the trait and molecular markers can identify genes in close proximity to the trait (Mauricio, 2001). Advantages of QTL mapping are that limited knowledge of the genome is necessary and producing bi-parental populations is relatively rapid (Fig. 3A). Recombinant inbred lines (RILs) can be produced, for example, from a segregating F_2_ generation through rounds of self-fertilization and so generate an immortalized population that can be genotyped once but phenotyped repeatedly. This is especially useful for heritability estimates and mapping QTL in different environments or years (Broman, 2005). Due to considerable differences in the biochemistry and physiology of C_3_ and C_4_ plants, if mapping populations derived from C_3_ and C_4_ parents of *Atriplex*, *Alloteropsis*, or *Flaveria* were generated, QTL mapping could probably associate genes with a wide variety of C_4_ phenotypes. *Alloteropsis semialata* could be of particular interest here because of the presence of both C_3_ and C_4_ subspecies that hybridize to produce offspring with intermediate characteristics (Bianconi *et al.*, 2021, Preprint). As self-fertilization is also possible, a population of RILs could be designed specifically for the investigation of C_4_ traits. High-throughput phenotyping combined with the convoluted neural network Mask R-CNN (He *et al.*, 2017) has been used for QTL mapping of C_4_-relevant traits in biparental populations. This allowed rapid assessment of thousands of images and identification of QTL for stomatal traits such as size and density (Xie *et al.*, 2021).

Although QTL mapping is used extensively, its power is limited if the trait is responsive to the environment and so has low heritability. The heritability of many C_4_ traits remains poorly understood, but there is growing evidence that variations in CO_2_ fixation processes and leaf anatomy exist (Table 1) and so estimates of heritability of such C_4_ traits should be possible. Given the complexity of photosynthesis, its ability to respond to the environment, and temporal variation in its efficiency, it is highly likely that low-heritability traits will be encountered (Flood *et al.*, 2016). Although traits with low heritability can be investigated using highly controlled environments, highly inbred populations in combination with high-density marker systems are necessary to capture the multiple small-effect QTL contributing to the low-heritability trait of interest. An alternative approach involves genome-wide association studies (GWAS) or linkage disequilibrium (LD) mapping, which identifies markers such as single nucleotide polymorphisms (SNPs) that are in LD with the phenotype of interest (Tam *et al.*, 2019). GWAS does not require a segregating population but rather uses many diverse accessions that represent thousands of years of recombination to capture multiple alleles, allowing marker groups (haplotypes) to be identified in close association with causal loci. Additionally, it has the advantage of being feasible for obligate outcrossers. In order to work successfully, GWAS requires many markers since it relies on LD decay (Mackay and Powell, 2007) and, as pedigrees are unknown, physical maps are also needed. Although population structure increases the number of false positives derived from GWAS (Korte and Farlow, 2013), this is increasingly being overcome by statistical modelling (Cortes *et al.*, 2021). GWAS has identified QTL associated with photosynthetic performance during chilling in maize (Strigens *et al.*, 2013) and sorghum (Ortiz *et al.*, 2017). More recently, a sorghum diversity panel of 756 African accessions was described (Faye *et al.*, 2021) and a diverse 869 line panel (Valluru *et al.*, 2019) was subjected to GWAS to identify genes controlling stomatal conductance and water use efficiency (Ferguson *et al.*, 2021; Pignon *et al.*, 2021). The latter two studies used transcriptome data to allow transcriptome-wide association as well as GWAS (reviewed by Wainberg *et al.*, 2019) to increase the likelihood of identifying candidate genes. Association mapping has also been used to study the light-dependent reactions of photosynthesis (van Bezouw *et al.*, 2019) but, to our knowledge, QTL determining differences in C_4_ carbon fixation or Kranz anatomy have not yet been identified. The sorghum and maize mapping panels present an avenue through which targeted phenotyping of C_4_-specific traits could be used to identify genes responsible for the C_4_ syndrome. For example, if a gene controlling bundle sheath cell size was identified through mapping in maize or sorghum, this could then be introduced in a C_3_ crop such as rice to determine whether this allowed engineering of this trait.

**Table 1.** Summary of publications documenting intraspecific variation in traits relevant to C_4_ photosynthesis-associated traits

Species	Varying trait	Reference
*Alloteropsis semialata* (C_4_ accessions)	Abundance of *PEPC* and *PEPCK* transcripts	Dunning *et al.* (2017)
	PEPC content Carbon isotope discrimination Mesophyll cell size Bundle sheath cell size Leaf physiology	Lundgren *et al.* (2016)
*Gynandropsis gynandra*	C_4_ transcript abundance, physiology, and leaf morphology	Reeves *et al.* (2018)
*Panicum coloratum*	Chloroplast location Bundle sheath suberization	Ohsugi and Murata (1985)
*Setaria italica*	Carbon isotope Differing intensities of green’	Lightfoot *et al.* (2016)
*Sorghum bicolor*	Net assimilation rate	Kataria and Guruprasad (2012)
*Zea mays*	*CA* transcript abundance	Zhang *et al.* (2015)
*Zea mays*	CA, PEPC, and Rubisco activity Net assimilation rate Interveinal distance Mesophyll thickness Maximum assimilation rate	Kolbe and Cousins (2018)
	CA, PEPC, and Rubisco activity C_4_ transcript abundance Carbon isotope	Kolbe *et al.* (2018)
	Vein density Gas exchange traits PEPC, NADP-ME, PEPCK, and Rubisco activity	Yabiku and Ueno (2017)

CA, carbonic anhydrase, NADP-ME; NADP-dependent malic enzyme; PEPC, phospho*enol*pyruvate carboxylase; PEPCK, phospho*enol*pyruvate carboxykinase.

Association mapping can be combined with specific breeding pedigrees to capture multiple recombination events, account for population structure, and so allow higher resolution mapping. These include nested-association mapping (NAM) and multiparent advanced generation inter-crossing (MAGIC) population designs. Both address issues with GWAS and capture more allelic variation than bi-parental populations. Whilst allelic diversity is reduced in these multiparent designs compared with GWAS, linkage mapping as well as association mapping are possible, and this is particularly useful when a physical map is not available (Broman *et al.*, 2018). Thus, NAM and MAGIC are currently particularly relevant for C_4_ photosynthesis because although annotated genome sequences are being developed for, for example, *Alloteropsis* sp., *Flaveria* sp., and *G. gynandra*, complete and well-annotated genomes for many C_4_ model species have not yet been developed. The NAM design involves crossing one recurrent parent with many other accessions. Progeny from each cross are initially bulked and then self-fertilized for multiple generations, leading to multiple RIL families (one family per unique founder) that then constitute the final NAM population (Yu *et al.*, 2008; McMullen *et al.*, 2009). At least two NAM populations exist for maize (Yu *et al.*, 2008; Chen *et al.*, 2019) and, as mentioned above, significant variation for δ^13^C as well as CA, PEPC, and Rubisco activities has been reported in the founder lines (Zhang *et al.*, 2015; Kolbe *et al.*, 2018; Twohey *et al.*, 2019). Despite this, QTL for these traits have to our knowledge not yet been determined. A sorghum NAM population has been used in conjunction with an association panel to identify QTL for grain filling (Tao *et al.*, 2020). NAM populations offer the chance to study an extremely divergent line, such as a pre-domesticated species in the background of a stable population. This has been done with teosinte and maize as the recurrent parent (Chen *et al.*, 2019). Given the noted differences in maize and teosinte photosynthetic capacity (Yabiku and Ueno, 2017), this offers an interesting resource to map traits that differ between these species.

The MAGIC design also relies on homozygous founder lines that differ in traits of interest. Intercrossing for multiple generations allows segregating populations to be formed consisting of lines that capture the founder genomes in unique recombinants (Fig. 3B). Such segregating lines then undergo self-fertilization for several generations to generate RILs that capture multiple allele combinations from the various parents (Cavanagh *et al.*, 2008). With MAGIC, haplotype diversity is not limited by the use of a single recurrent parent (Ladejobi *et al.*, 2016) and, although the MAGIC design requires large amounts of hybridization and significant time to produce the final population (Huang *et al.*, 2015; Pascual *et al.*, 2015; Ongom and Ejeta, 2017; Mahan *et al.*, 2018), simplified strategies can be implemented (Stadlmeier *et al.*, 2018). In the context of C_4_ photosynthesis, MAGIC RILs are available for maize and sorghum (Dell’Acqua *et al.*, 2015; Ongom and Ejeta, 2017; Mahan *et al.*, 2018; Butrón *et al.*, 2019). Additionally, transcriptome data exist for the founders of one maize MAGIC population (Dell’Acqua *et al.*, 2015) and 94 of the MAGIC RILs (Baute *et al.*, 2016). Should these RILs possess variation in activity of C_4_ enzymes or components of Kranz anatomy, QTL could be identified. To our knowledge, there is currently no MAGIC population available for a C_4_ dicotyledon, nor a mapping panel designed explicitly to map C_4_ photosynthetic traits. As variation in C_4_ traits has been reported in *A. semialata* and *G. gynandra* (Lundgren *et al.*, 2016; Reeves *et al.*, 2018) and they can be crossed (Sogbohossou *et al.*, 2018; Bianconi *et al.*, 2020), mapping resources in these species would be useful.

**Fig. 4.**
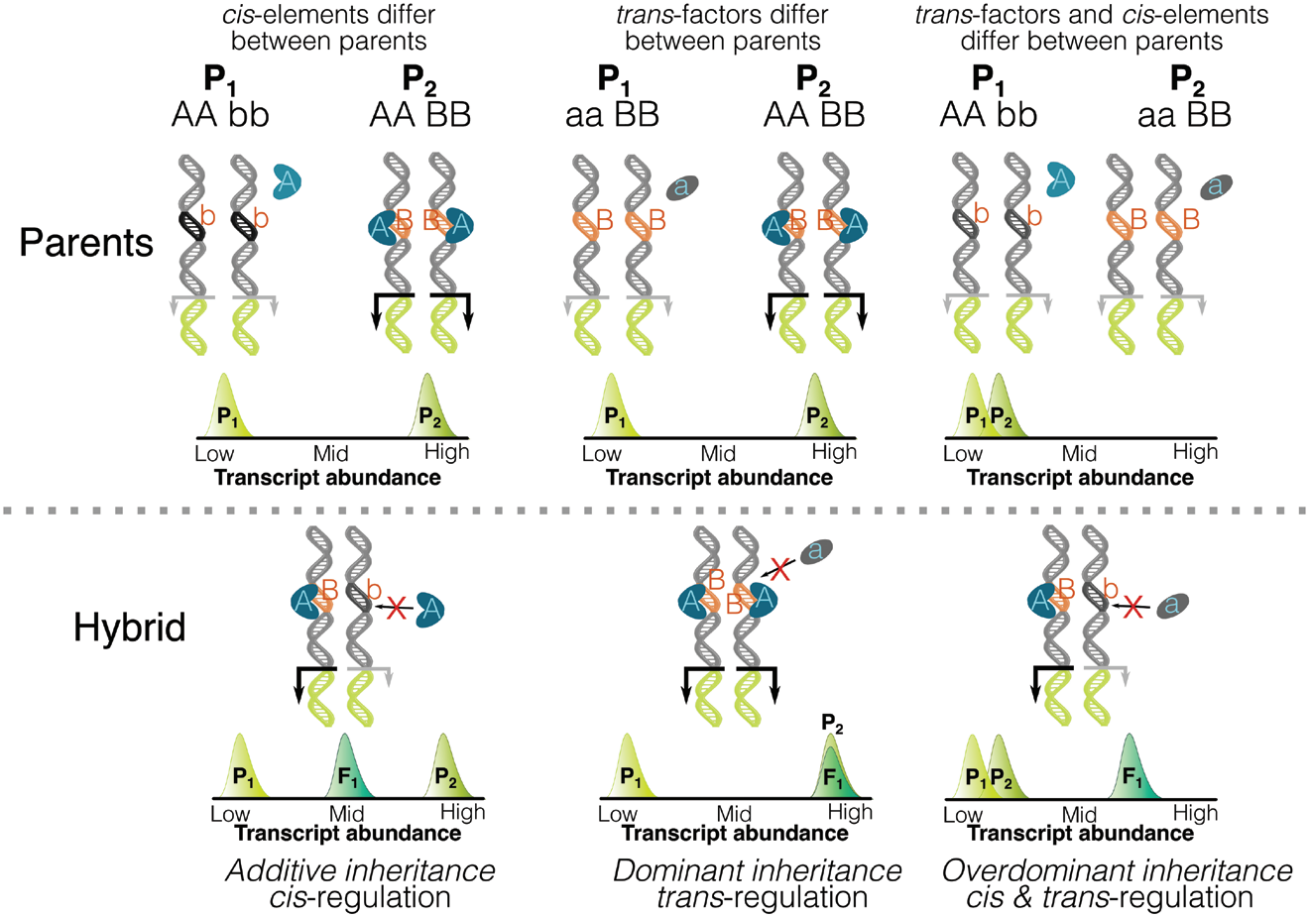
Using breeding to understand the molecular basis of C_4_ gene regulation. Parental populations that differ in transcript abundance can be due to multiple genetic effects that can be parsed by quantitative genetics. A simplified two loci model where one locus is a *cis*-element and the other an activating *trans*-factor is presented to illustrate how the molecular basis underpinning variations in gene expression can be determined by inheritance of gene expression in F_1_ hybrids. If expression of a gene is controlled by changes in *cis*-regulation between parents, offspring exhibit additive expression patterns. If variation in expression is due to changes *in trans* between parents, then offspring exhibit dominance deviation towards one parent. Lastly, if differences in gene expression between parents is due to both *cis* and *trans* factors, offspring demonstrate heterosis or overdominance.

Once a QTL is identified using any of the above population types, fine mapping enables causative genes to be identified (Hormozdiari *et al.*, 2014; Tam *et al.*, 2019). Parsing C_4_ photosynthesis into individual components, such genes controlling C_4_ enzyme activity or bundle sheath cell size (Dunning *et al.*, 2017) are identified by different phenotyping techniques which, combined with fine mapping, could identify additional genes required for C_4_ photosynthesis. Exploiting the high degree of natural variation among C_3_ and C_4_ species will enable genome-wide associations to help map critical photosynthesis regulators. Furthermore, inferences into the inheritance of C_4_ components such as cell-specific gene expression can be parsed even without proper segregation or recombination in C_3_ and C_4_ hybrids (Fig. 4). While such methods cannot identify QTL, they can at least establish broad modes of inheritance (Charlesworth and Willis, 2009). For example, sterile F_1_ populations derived from C_3_ and C_4_ parents that show altered transcript abundance or cellular localization of C_4_ enzymes can provide insight into whether genes are controlled in *cis*, *trans*, or a combination of both mechanisms, and whether these mechanisms are functioning in an activating or repressive manner (Fig. 4). This technique has been deployed in F_1_ hybrids derived from a cross between the C_3_–C_4_ intermediate *Moricandia arvensis* and the C_3_*M. moricandiodes* to show that *cis*-regulation dominates control of photosynthetic and anatomical phenotypes (Lin *et al.*, 2021, Preprint). Information from such studies could inform mapping strategies and marker placement for associations.

In summary, in order to modify C_3_ leaves to perform C_4_ photosynthesis, an improved understanding of C_4_ anatomy, cell biology, and biochemistry is needed. Wide hybridization by either sexual or asexual means to recombine interspecific variation found in C_3_ and C_4_ species or intraspecific photosynthetic variation in C_4_ species, combined with mapping populations and high-throughput phenotyping, should facilitate a better understanding of C_4_ photosynthesis. Quantitative genetics then offer robust methods to better understand the regulatory mechanisms behind these traits. Applying these techniques therefore promises to enhance photosynthetic efficiency of C_3_ and C_4_ crops and so contribute to a more robust world agriculture in the future.

## References

[CIT0001] Ahmad F , Comeau A. 1990. Wheat × pearl millet hybridization: consequence and potential. Euphytica 50, 181–190.

[CIT0002] Akhani H , Ghasemkhani M, Chuong SDX, Edwards GE. 2008. Occurrence and forms of Kranz anatomy in photosynthetic organs and characterization of NAD-ME subtype C_4_ photosynthesis in *Blepharis ciliaris* (L.) B. L. Burtt (Acanthaceae). Journal of Experimental Botany 59, 1755–1765. 18440932 10.1093/jxb/ern020

[CIT0003] Apel P , Bauwe H, Bassüner B, Maass I. 1988. Photosynthetic properties of *Flaveria cronquistii, F. palmeri*, and hybrids between them. Biochemie und Physiologie der Pflanzen 183, 291–299.

[CIT0004] Baute J , Herman D, Coppens F, De Block J, Slabbinck B, Acqua MD, Pè ME, Maere S, Nelissen H, Inzé D. 2016. Combined large-scale phenotyping and transcriptomics in maize reveals a robust growth regulatory network. Plant Physiology 170, 1848–1867. 26754667 10.1104/pp.15.01883 PMC4775144

[CIT0005] Bianconi ME , Dunning LT, Curran EV, et al. 2020. Contrasted histories of organelle and nuclear genomes underlying physiological diversification in a grass species. Proceedings of the Royal Society B: Biological Sciences 287, 20201960. 10.1098/rspb.2020.1960 PMC7735283 33171085

[CIT0006] Bianconi ME , Sotelo G, Curran EV, Milenkovic V, Samaritani E, Dunning LT, Osborne CP, Christin P. 2021. Upregulation of C_4_ characteristics does not consistently improve photosynthetic performance in intraspecific hybrids of a grass. bioRxiv. doi:10.1101/2021.08.10.455822. [Preprint]. PMC9314825 35201618

[CIT0007] Björkman O , Gauhl E, Nobs M. 1969. Comparative studies of *Atriplex* species with and without β-carboxylation photosynthesis. Carnegie Institution of Washington Yearbook 68, 620–633.

[CIT0008] Björkmann O , Nobs MA, Berry J. 1971. Further studies on hybrids beteen C_3_ and C_4_ species of *Atriplex*. Carnegie Institiute of Washington Annual Report 70, 507–511.

[CIT0009] Botha CEJ. 1992. Plasmodesmatal distribution, structure and frequency in relation to assimilation in C_3_ and C_4_ grasses in southern Africa. Planta 187, 348–358. 24178075 10.1007/BF00195658

[CIT0010] Bouton JH , Brown RH, Evans PT, Jernstedt JA. 1986. Photosynthesis, leaf anatomy, and morphology of progeny from hybrids between C_3_ and C_3_/C_4_*Panicum* species. Plant Physiology 80, 487–492. 16664649 10.1104/pp.80.2.487 PMC1075141

[CIT0011] Bowes G , Ogren WL, Hageman RH. 1971. Phosphoglycolate production catalyzed by ribulose diphosphate carboxylase. Biochemical and Biophysical Research Communications 45, 716–722. 4331471 10.1016/0006-291x(71)90475-x

[CIT0012] Broman KW. 2005. The genomes of recombinant inbred lines. Genetics 169, 1133–1146. 15545647 10.1534/genetics.104.035212 PMC1449115

[CIT0013] Broman KW , Gatti DM, Simecek P, Furlotte NA, Prins P, Sen S, Yandell BS, Churchill GA. 2018. R/qtl2: software for mapping quantitative trait loci with high-dimensional data and for mapping quantitative trait loci with high-dimensional data and multiparent populations high-dimensional data and multiparent populations. Genetics 211, 495–502. 30591514 10.1534/genetics.118.301595 PMC6366910

[CIT0014] Brown HR , Bouton JH. 1993. Interspecific hybrids between photosynthetic types. Annual Review of Plant Physiology 44, 435–436.

[CIT0015] Brown RH , Bassett CL, Cameron RG, Evans PT, Bouton JH, Black CC, Sternberg LO, Deniro MJ. 1986. Photosynthesis of F_1_ hybrids between C_4_ and C_3_–C_4_ species of *Flaveria*. Plant Physiology 82, 211–217. 16664994 10.1104/pp.82.1.211 PMC1056091

[CIT0016] Brown RH , Byrd GT, Black CC. 1992. Degree of C_4_ photosynthesis in C_4_ and C_3_–C_4_*Flaveria* species and their hybrids: II. Inhibition of apparent photosynthesis by a phosphoenolpyruvate carboxylase inhibitor. Plant Physiology 100, 947–950. 16653080 10.1104/pp.100.2.947 PMC1075648

[CIT0017] Butrón A , Santiago R, Cao A, Samayoa LF, Malvar RA. 2019. QTLs for resistance to *Fusarium* ear rot in a Multiparent Advanced Generation Intercross (MAGIC) maize population. Plant Disease 109, 897–904. 10.1094/PDIS-09-18-1669-RE 30856072

[CIT0018] Calvin M , Benson AA. 1948. The path of carbon in photosynthesis. Encyclopedia of the Environment 107, 476–480. 10.1126/science.107.2784.476 17760010

[CIT0019] Cameron RG , Bassett CL, Bouton JH, Brown RH. 1989. Transfer of C_4_ photosynthetic characters through hybridization of *Flaveria* species. Plant Physiology 90, 1538–1545. 16666962 10.1104/pp.90.4.1538 PMC1061922

[CIT0020] Carlson PS , Smith HH, Dearing R. 1972. Parasexual interspecific plant hybridization. Proceedings of the National Academy of Sciences, USA 69, 2292–2294. 10.1073/pnas.69.8.2292 PMC426920 16592009

[CIT0021] Cavanagh C , Morell M, Mackay I, Powell W. 2008. From mutations to MAGIC: resources for gene discovery, validation and delivery in crop plants. Current Opinion in Plant Biology 11, 215–221. 18295532 10.1016/j.pbi.2008.01.002

[CIT0022] Charlesworth D , Willis JH. 2009. The genetics of inbreeding depression. Nature Reviews. Genetics 10, 783–796. 10.1038/nrg2664 19834483

[CIT0023] Chen Q , Yang CJ, York AM, et al. 2019. TeoNAM: a nested association mapping population for domestication and agronomic trait analysis in maize. Genetics 213, 1065–1078. 31481533 10.1534/genetics.119.302594 PMC6827374

[CIT0024] Choudhury SD , Samal A, Awada T. 2019. Leveraging image analysis for high-throughput. Plant Phenotyping 10, 1–8. 10.3389/fpls.2019.00508 PMC6491831 31068958

[CIT0025] Cortes LT , Zhang Z, Yu J. 2021. Status and prospects of genome-wide association studies in plants. Plant Genome 14, 1–17. 10.1002/tpg2.20077 PMC12806871 33442955

[CIT0026] Danila FR , Quick WP, White RG, Furbank RT. 2016. The metabolite pathway between bundle sheath and mesophyll: quantification of plasmodesmata in leaves of C_3_ and C_4_ monocots. The Plant Cell 28, 1461–1471. 27288224 10.1105/tpc.16.00155 PMC4944413

[CIT0027] Dell’Acqua M , Gatti DM, Pea G, et al. 2015. Genetic properties of the MAGIC maize population: a new platform for high definition QTL mapping in *Zea mays*. Genome Biology 16, 1–23. 26357913 10.1186/s13059-015-0716-z PMC4566846

[CIT0028] Dunning LT , Lundgren MR, Moreno-Villena JJ, Namaganda M, Edwards EJ, Nosil P, Osborne CP, Christin PA. 2017. Introgression and repeated co-option facilitated the recurrent emergence of C_4_ photosynthesis among close relatives. Evolution 71, 1541–1555. 28395112 10.1111/evo.13250 PMC5488178

[CIT0029] Edwards EJ , Smith SA. 2010. Phylogenetic analyses reveal the shady history of C_4_ grasses. Proceedings of the National Academy of Sciences, USA 107, 2532–2537. 10.1073/pnas.0909672107 PMC2823882 20142480

[CIT0030] Edwards GE , Voznesenskaya EV. 2011. C_4_ phtosynthesis: Kranz forms and single-cell C_4_ in terrestrial plants. In: Raghavendra AS, Sage RF, eds. C_4_ photosynthesis and related CO_2_ concentrating mechanisms. Dordrecht: Springer, 29–61.

[CIT0031] Evans DA. 1983. Agricultural applications of plant protoplast fusion. Nature Biotechnology 1, 253–261.

[CIT0032] Faye JM , Maina F, Akata EA, et al. 2021. A genomics resource for genetics, physiology, and breeding of West African sorghum. Plant Genome 14, 1–18. 10.1002/tpg2.20075 PMC12807439 33818011

[CIT0033] Ferguson JN , Fernandes SB, Monier B, et al. 2021. Machine leaning enabled phenotyping for GWAS and TWAS of WUE traits in 869 field-grown sorghum accessions. Plant Physiology 187, 1481–1500. 34618065 10.1093/plphys/kiab346 PMC9040483

[CIT0034] Flood PJ , Kruijer W, Schnabel SK, Schoor R, Jalink H, Snel JFH, Harbinson J, Aarts MGM. 2016. Phenomics for photosynthesis, growth and reflectance in *Arabidopsis thaliana* reveals circadian and long-term fluctuations in heritability. Plant Methods 12, 1–14. 26884806 10.1186/s13007-016-0113-y PMC4754911

[CIT0035] Furbank RT. 2011. Evolution of the C_4_ photosynthetic mechanism: are there really three C_4_ acid decarboxylation types? Journal of Experimental Botany 62, 3103–3108. 21511901 10.1093/jxb/err080

[CIT0036] Gernand D , Rutten T, Varshney A, Rubtsova M, Prodanovic S, Brüß C, Kumlehn J, Matzk F, Houben A. 2005. Uniparental chromosome elimination at mitosis and interphase in wheat and pearl millet crosses involves micronucleus formation, progressive heterochromatinization, and DNA fragmentation. The Plant Cell 17, 2431–2438. 16055632 10.1105/tpc.105.034249 PMC1197425

[CIT0037] Hatch M , Kagawa T, Craig S. 1975. Subdivision of C_4_-pathway species based on differing C_4_ acid decarboxylating systems and ultrastructural features. Functional Plant Biology 2, 111.

[CIT0038] He K , Gkioxari G, Dollar P, Girshick R. 2017. Mask R-CNN. In: IEEE International Conference on Computer Vision, 2980–2988.

[CIT0039] Holaday AS , Brown RH, Bartlett JM, Sandlin EA, Jackson RC. 1988. Enzymic and photosynthetic characteristics of reciprocal F_1_ hybrids of *Flaveria pringlei* (C_3_) and *Flaveria brownii* (C_4_-like species). Plant Physiology 87, 484–490. 16666169 10.1104/pp.87.2.484 PMC1054779

[CIT0040] Hormozdiari F , Kostem E, Kang EY, Pasaniuc B, Eskin E. 2014. Identifying causal variants at loci with multiple signals of association. Genetics 198, 497–508. 25104515 10.1534/genetics.114.167908 PMC4196608

[CIT0041] Huang BE , Verbyla KL, Verbyla AP, Raghavan C, Singh VK, Gaur P, Leung H, Varshney RK, Cavanagh CR. 2015. MAGIC populations in crops: current status and future prospects. Theoretical and Applied Genetics 128, 999–1017. 25855139 10.1007/s00122-015-2506-0

[CIT0042] Ishii T , Ueda T, Tanaka H, Tsujimoto H. 2010. Chromosome elimination by wide hybridization between Triticeae or oat plant and pearl millet: pearl millet chromosome dynamics in hybrid embryo cells. Chromosome Research 18, 821–831. 20953694 10.1007/s10577-010-9158-3

[CIT0043] Ishii T , Tanaka H, Eltayeb AE, Tsujimoto H. 2013. Wide hybridization between oat and pearl millet belonging to different subfamilies of Poaceae. Plant Reproduction 26, 25–32.

[CIT0044] Ishii T , Sunamura N, Matsumoto A, Eltayeb AE, Tsujimoto H. 2015. Preferential recruitment of the maternal centromere-specific histone H3 (CENH3) in oat (*Avena sativa* L.) × pearl millet (*Pennisetum glaucum* L.) hybrid embryos. Chromosome Research 23, 709–718. 26134441 10.1007/s10577-015-9477-5

[CIT0045] Jin W , Melo JR, Nagaki K, Talbert PB, Henikoff S, Dawe RK, Jiang J. 2004. Maize centromeres: organization and functional adaptation in the genetic background of oat. The Plant Cell 16, 571–581. 14973167 10.1105/tpc.018937 PMC385273

[CIT0046] Kadereit G , Borsch T, Weising K, Freitag H. 2003. Phylogeny of Amaranthaceae and Chenopodiaceae and the evolution of C_4_ photosynthesis. International Journal of Plant Sciences 164, 959–986.

[CIT0047] Kataria S , Guruprasad KN. 2012. Intraspecific variations in growth, yield and photosynthesis of sorghum varieties to ambient UV (280–400nm) radiation. Plant Science 196, 85–92. 23017902 10.1016/j.plantsci.2012.07.011

[CIT0048] Kolbe AR , Cousins AB. 2018. Mesophyll conductance in *Zea mays* responds transiently to CO_2_ availability: implications for transpiration efficiency in C_4_ crops. New Phytologist 217, 1463–1474. 29220090 10.1111/nph.14942

[CIT0049] Kolbe AR , Studer AJ, Cousins AB. 2018. Biochemical and transcriptomic analysis of maize diversity to elucidate drivers of leaf carbon isotope composition. Functional Plant Biology 45, 489–500. 32290988 10.1071/FP17265

[CIT0050] Korte A , Farlow A. 2013. The advantages and limitations of trait analysis with GWAS: a review. Plant Methods 9, 291. 10.1186/1746-4811-9-29 PMC3750305 23876160

[CIT0051] Koteyeva NK , Voznesenskaya EV, Roalson EH, Edwards GE. 2011. Diversity in forms of C_4_ in the genus *Cleome* (Cleomaceae). Annals of Botany 107, 269–283. 21147832 10.1093/aob/mcq239 PMC3025737

[CIT0052] Kynast RG , Riera-Lizarazu O, Vales MI, et al. 2001. A complete set of maize individual chromosome additions to the oat genome. Plant Physiology 125, 1216–1227. 11244103 10.1104/pp.125.3.1216 PMC65602

[CIT0053] Kynast RG , Okagaki RJ, Galatowitsch MW, Granath SR, Jacobs MS, Stec AO, Rines HW, Phillips RL. 2004. Dissecting the maize genome by using chromosome addition and radiation hybrid lines. Proceedings of the National Academy of Sciences, USA 101, 9921–9926. 10.1073/pnas.0403421101 PMC470774 15197265

[CIT0054] Ladejobi O , Elderfield J, Gardner KA, Gaynor RC, Hickey J, Hibberd JM, Mackay IJ, Bentley AR. 2016. Maximizing the potential of multi-parental crop populations. Applied and Translational Genomics 11, 9–17. 28018845 10.1016/j.atg.2016.10.002 PMC5167364

[CIT0055] Laurie DA , Bennett MD. 1986. Wheat × maize hybridization. Canadian Journal of Genetics and Cytology 28, 313–316.

[CIT0056] Laurie DA , Bennett MD. 1989. The timing of chromosome elimination in hexaploid wheat × maize crosses. Genome 32, 953–961.

[CIT0057] Laurie DA , O’Donoughue LS, Bennett MD. 1990. Wheat × maize and other wide sexual hybrids: their potential for genetic manipulation and crop improvement. In: Gustafson JP, ed. Gene manipulation in plant improvement II. Boston, MA: Springer, 95–126.

[CIT0058] Leary MHO. 1988. Carbon isotopes in photosynthesis. BioScience 38, 328–336.

[CIT0059] Lightfoot E , Przelomska N, Craven M, O Connell TC, He L, Hunt HV, Jones MK. 2016. Intraspecific carbon and nitrogen isotopic variability in foxtail millet (*Setaria italica*). Rapid Communications in Mass Spectrometry 30, 1475–1487. 27321835 10.1002/rcm.7583

[CIT0060] Lin M , Schlüter U, Stich B, Weber APM. 2021. Cis -regulatory divergence underpins the evolution of C_3_–C_4_ intermediate photosynthesis in *Moricandia*. bioRxiv. doi:10.1101/2021.05.10.443365. [Preprint].

[CIT0061] Lundgren MR , Christin PA, Escobar EG, Ripley BS, Besnard G, Long CM, Hattersley PW, Ellis RP, Leegood RC, Osborne CP. 2016. Evolutionary implications of C_3_–C_4_ intermediates in the grass *Alloteropsis semialata*. Plant, Cell & Environment 39, 1874–1885. 10.1111/pce.12665 26524631

[CIT0062] Mackay I , Powell W. 2007. Methods for linkage disequilibrium mapping in crops. Trends in Plant Science 12, 57–63. 17224302 10.1016/j.tplants.2006.12.001

[CIT0063] Mahan AL , Murray SC, Klein PE. 2018. Four-Parent Maize (FPM) population: development and phenotypic characterization. Crop Science 58, 1106–1117.

[CIT0064] Mauricio R. 2001. Mapping quantitative trait loci in plants: uses and caveats for evolutionary biology. Nature Reviews. Genetics 2, 370–381. 10.1038/35072085 11331903

[CIT0065] McMullen MD , Kresovich S, Villeda HS, et al. 2009. Genetic properties of the maize nested association mapping population. Science 325, 737–740. 19661427 10.1126/science.1174320

[CIT0066] Mertz RA , Brutnell TP. 2014. Bundle sheath suberization in grass leaves: multiple barriers to characterization. Journal of Experimental Botany 65, 3371–3380. 24659485 10.1093/jxb/eru108

[CIT0067] Muhaidat R , Sage RF, Dengler NG. 2007. Diversity of Kranz anatomy and biochemistry in C_4_ eudicots. American Journal of Botany 94, 362–381. 21636407 10.3732/ajb.94.3.362

[CIT0068] Nobs MA , Björkmann O, Pearcy RW, Boynton JE. 1970. Hybrids between *Atriplex* species with and without beta-carboxylation photosynthesis. Carnegie Institution of Washington Yearbook 69, 617–662.

[CIT0069] Oakley JC , Sultmanis S, Stinson CR, Sage TL, Sage RF. 2014. Comparative studies of C_3_ and C_4_*Atriplex* hybrids in the genomics era: physiological assessments. Journal of Experimental Botany 65, 3637–3647. 24675672 10.1093/jxb/eru106 PMC4085961

[CIT0070] Ohsugi R , Murata T. 1985. C_4_ photosynthetic characteristics of *Panicum* species in the Dichotomiflora group. Japan Agricultural Research Quarterly 19, 125–131.

[CIT0071] Omoto E , Taniguchi M, Miyake H. 2012. Adaptation responses in C_4_ photosynthesis of maize under salinity. Journal of Plant Physiology 169, 469–477. 22209164 10.1016/j.jplph.2011.11.009

[CIT0072] Ongom PO , Ejeta G. 2017. Mating design and genetic structure of a multi-parent advanced generation intercross (MAGIC) population of sorghum (*Sorghum bicolor* L. Moench). G3 8, 331–341. 10.1534/g3.117.300248 PMC5765360 29150594

[CIT0073] Ortiz D , Hu J, Salas Fernandez MG. 2017. Genetic architecture of photosynthesis in *Sorghum bicolor* under non-stress and cold stress conditions. Journal of Experimental Botany 68, 4545–4557. 28981780 10.1093/jxb/erx276 PMC5853419

[CIT0074] Pascual L , Desplat N, Huang BE, Desgroux A, Bruguier L, Bouchet JP, Le QH, Chauchard B, Verschave P, Causse M. 2015. Potential of a tomato MAGIC population to decipher the genetic control of quantitative traits and detect causal variants in the resequencing era. Plant Biotechnology Journal 13, 565–577. 25382275 10.1111/pbi.12282

[CIT0075] Peter G , Katinas L. 2003. A new type of Kranz anatomy in Asteraceae. Australian Journal of Botany 51, 217–226.

[CIT0076] Pignon CP , Fernandes SB, Valluru R, Bandillo N, Lozano R, Buckler E, Gore MA, Long SP, Brown PJ, Leakey ADB. 2021. Phenotyping stomatal closure by thermal imaging for GWAS and TWAS of water use efficiency-related genes. Plant Physiology doi: 10.1093/plphys/kiab395. PMC8644692 34618072

[CIT0077] Portis AR , Parry MAJ. 2007. Discoveries in Rubisco (Ribulose 1,5-bisphosphate carboxylase/oxygenase): a historical perspective. Photosynthesis Research 94, 121–143. 17665149 10.1007/s11120-007-9225-6

[CIT0078] Prendergast HDV. 1987. Structural, biochemical and geographical relationships in Australian C4 grasses. PhD thesis, Australian National University.

[CIT0079] Reeves G , Singh P, Rossberg TA, Sogbohossou EOD, Schranz ME, Hibberd JM. 2018. Natural variation within a species for traits underpinning C_4_ photosynthesis. Plant Physiology 177, 504–512. 29678862 10.1104/pp.18.00168 PMC6001323

[CIT0080] Rikiishi K , Oguro H, Samejima M, Sugiyama T, Hinata K. 1988. C_4_-like plants derived from a cross (*Atriplex rosea* (C_4_) × *A. patula* (C_3_)) × *A. rosea*. Japanese Journal of Breeding 38, 397–408.

[CIT0081] Sage RF. 2016. A portrait of the C_4_ photosynthetic family on the 50th anniversary of its discovery: species number, evolutionary lineages, and hall of fame. Journal of Experimental Botany 67, 4039–4056. 27053721 10.1093/jxb/erw156

[CIT0082] Sage RF , Stata M. 2015. Photosynthetic diversity meets biodiversity: the C_4_ plant example. Journal of Plant Physiology 172, 104–119. 25264020 10.1016/j.jplph.2014.07.024

[CIT0083] Sage RF , Christin P-A, Edwards EJ. 2011. The C_4_ plant lineages of planet Earth. Journal of Experimental Botany 62, 3155–3169. 21414957 10.1093/jxb/err048

[CIT0084] Sales CRG , Ribeiro RV, Hayashi AH, Marchiori PER, Silva KI, Martins MO, Silveira JAG, Silveira NM, Machado EC. 2018. Flexibility of C_4_ decarboxylation and photosynthetic plasticity in sugarcane plants under shading. Environmental and Experimental Botany 149, 34–42.

[CIT0085] Sedelnikova OV , Hughes TE, Langdale JA. 2018. Understanding the genetic basis of C_4_ Kranz anatomy with a view to engineering C_3_ crops. Annual Review of Genetics 52, 249–270. 10.1146/annurev-genet-120417-031217 30208293

[CIT0086] Sharkey TD. 1988. Estimating the rate of photorespiration in leaves. Physiologia Plantarum 73, 147–152.

[CIT0087] Sharwood RE , Sonawane BV, Ghannoum O. 2014. Photosynthetic flexibility in maize exposed to salinity and shade. Journal of Experimental Botany 65, 3715–3724. 24692650 10.1093/jxb/eru130 PMC4085963

[CIT0088] Sogbohossou EOD , Achigan-Dako EG, Maundu P, Solberg S, Deguenon EMS, Mumm RH, Hale I, Van Deynze A, Schranz ME. 2018. A roadmap for breeding orphan leafy vegetable species: a case study of *Gynandropsis gynandra* (Cleomaceae). Horticulture Research 5, 1–15. 29423232 10.1038/s41438-017-0001-2 PMC5798814

[CIT0089] Stadlmeier M , Hartl L, Mohler V. 2018. Usefulness of a multiparent advanced generation intercross population with a greatly reduced mating design for genetic studies in winter wheat. Frontiers in Plant Science 871, 1–12. 10.3389/fpls.2018.01825 PMC6291512 30574161

[CIT0090] Sternberg LDSL , Deniro MJ, Sloan ME, Black CC. 1986. Compensation point and isotopic characteristics of C_3_/C_4_ intermediates and hybrids in *Panicum*. Plant Physiology 80, 242–245. 16664590 10.1104/pp.80.1.242 PMC1075089

[CIT0091] Strigens A , Freitag NM, Gilbert X, Grieder C, Riedelsheimer C, Schrag TA, Messmer R, Melchinger AE. 2013. Association mapping for chilling tolerance in elite flint and dent maize inbred lines evaluated in growth chamber and field experiments. Plant, Cell & Environment 36, 1871–1887. 10.1111/pce.12096 23488576

[CIT0092] Szarka B , Göntér I, Molnár-Láng M, Mórocz S, Dudits D. 2002. Mixing of maize and wheat genomic DNA by somatic hybridization in regenerated sterile maize plants. Theoretical and Applied Genetics 105, 1–7. 12582555 10.1007/s00122-002-0877-5

[CIT0093] Tam V , Patel N, Turcotte M, Bossé Y, Paré G, Meyre D. 2019. Benefits and limitations of genome-wide association studies. Nature Reviews. Genetics 20, 467–484. 10.1038/s41576-019-0127-1 31068683

[CIT0094] Tao Y , Zhao X, Wang X, Hathorn A, Hunt C, Cruickshank AW, Erik J, Godwin ID, Mace ES, Jordan DR. 2020. Large-scale GWAS in sorghum reveals common genetic control of grain size among cereals. Plant Biotechnology Journal 18, 1093–1105. 31659829 10.1111/pbi.13284 PMC7061873

[CIT0095] Terada R , Kyozuka J, Nishibayashi S, Shimamoto K. 1987. Plantlet regeneration from somatic hybrids of rice (*Oryza sativa* L.) and barnyard grass (*Echinochloa oryzicola* Vasing). Molecular & General Genetics 210, 39–43.

[CIT0096] The Angiosperm Phylogeny Group . 2016. An update of the Angiosperm Phylogeny Group classification for the orders and families of flowering plants: APG IV. Botanical Journal of the Linnean Society 181, 1–20.

[CIT0097] Tolley BJ , Sage TL, Langdale JA, Hibberd JM. 2012. Individual maize chromosomes in the C_3_ plant oat can increase bundle sheath cell size and vein density. Plant Physiology 159, 1418–1427. 22675083 10.1104/pp.112.200584 PMC3425187

[CIT0098] Twohey RJ III , Roberts LM, Studer AJ. 2019. Leaf stable carbon isotope composition reflects transpiration efficiency in *Zea mays*. The Plant Journal 97, 475–484. 30351458 10.1111/tpj.14135

[CIT0099] Valluru R , Gazave EE, Fernandes SB, Ferguson JN, Lozano R, Hirannaiah P, Zuo T, Brown PJ, Leakey ADB, Gore MA. 2019. Deleterious mutation burden and its association with complex traits in sorghum (*Sorghum bicolor*). Genetics 211, 1075–1087. 30622134 10.1534/genetics.118.301742 PMC6404259

[CIT0100] van Bezouw RFHM , Keurentjes JJB, Harbinson J, Aarts MGM. 2019. Converging phenomics and genomics to study natural variation in plant photosynthetic efficiency. The Plant Journal 97, 112–133. 30548574 10.1111/tpj.14190 PMC6850172

[CIT0101] Voznesenskaya EV , Koteyeva NK, Chuong SDX, Ivanova AN, Barroca J, Craven LA, Edwards GE. 2007. Physiological, anatomical and biochemical characterisation of photosynthetic types in genus *Cleome* (Cleomaceae). Functional Plant Biology 34, 247–267. 32689352 10.1071/FP06287

[CIT0102] Voznesenskaya EV , Koteyeva NK, Edwards GE, Ocampo G. 2017. Unique photosynthetic phenotypes in *Portulaca* (Portulacaceae): C_3_–C_4_ intermediates and NAD-ME C_4_ species with Pilosoid-type Kranz anatomy. Journal of Experimental Botany 68, 225–239. 27986845 10.1093/jxb/erw393 PMC5853368

[CIT0103] Wainberg M , Sinnott-Armstrong N, Mancuso N, et al. 2019. Opportunities and challenges for transcriptome-wide association studies. Nature Genetics 51, 592–599. 30926968 10.1038/s41588-019-0385-z PMC6777347

[CIT0104] Wang K , Wu Y, Zhang W, Dawe RK, Jiang J. 2014. Maize centromeres expand and adopt a uniform size in the genetic background of oat. Genome Research 24, 107–116. 24100079 10.1101/gr.160887.113 PMC3875851

[CIT0105] Wang TB , Niizeki M. 1994. Somatic hybridization between *Zea mays* and *Triticum* sect. *tritirigia*. In: Bajaj YPS, ed. Biotechnology in agriculture and forestry. Berlin, Heidelberg: Springer Berlin Heidelberg, 99–111.

[CIT0106] Wang TB , Niizeki M, Harada T, Ishikawa R, Qian YQ, Saito K. 1993. Establishment of somatic hybrid cell lines between *Zea mays* L. (maize) and *Triticum* sect, *trititrigia* MacKey (trititrigia). Theoretical and Applied Genetics 86, 371–376. 24193485 10.1007/BF00222104

[CIT0107] Williams BP , Johnston IG, Covshoff S, Hibberd JM. 2013. Phenotypic landscape inference reveals multiple evolutionary paths to C_4_ photosynthesis. eLife 2, 1–19. 10.7554/eLife.00961 PMC3786385 24082995

[CIT0108] Xie J , Fernandes SB, Mayfield-Jones D, Erice G, Choi M, E Lipka A, Leakey ADB. 2021. Optical topometry and machine learning to rapidly phenotype stomatal patterning traits for maize QTL mapping. Plant Physiology 187, 1462–1480. 34618057 10.1093/plphys/kiab299 PMC8566313

[CIT0109] Xin HW , Sun JS, Yan QS, Zhang XQ. 1997. Plant regeneration from asymmetric somatic hybrids of *Oryza sativa* and *Panicum maximum*. Acta Botanica Sinica 39, 717–724.

[CIT0110] Xu C , Xia G, Zhi D, Xiang F, Chen H. 2003. Integration of maize nuclear and mitochondrial DNA into the wheat genome through somatic hybridization. Plant Science 165, 1001–1008.

[CIT0111] Yabiku T , Ueno O. 2017. Variations in physiological, biochemical, and structural traits of photosynthesis and resource use efficiency in maize and teosintes (NADP-ME-type C4). Plant Production Science 20, 448–458.

[CIT0112] Yin X , Struik PC. 2020. Viewpoints: Exploiting differences in the energy budget among C_4_ subtypes to improve crop productivity. New Phytologist 229, 2400–2409. 33067814 10.1111/nph.17011 PMC7894359

[CIT0113] Yu J , Holland JB, McMullen MD, Buckler ES. 2008. Genetic design and statistical power of nested association mapping in maize. Genetics 178, 539–551. 18202393 10.1534/genetics.107.074245 PMC2206100

[CIT0114] Zhang N , Gibon Y, Wallace JG, et al. 2015. Genome-wide association of carbon and nitrogen metabolism in the maize nested association mapping population. Plant Physiology 168, 575–583. 25918116 10.1104/pp.15.00025 PMC4453775

